# Psychological well-being and quality of life in visually impaired baseball players: An Italian national survey

**DOI:** 10.1371/journal.pone.0218124

**Published:** 2019-06-06

**Authors:** Daniela Mirandola, Marco Monaci, Guido Miccinesi, Alessia Vannuzzi, Eleonora Sgambati, Mirko Manetti, Mirca Marini

**Affiliations:** 1 Department of Experimental and Clinical Medicine, Section of Anatomy and Histology, University of Florence, Florence, Italy; 2 Oncological Network, Prevention and Research Institute (ISPRO), Florence, Italy; 3 Department of Biosciences and Territory, University of Molise, Pesche, Isernia, Italy; Università degli Studi di Perugia, ITALY

## Abstract

Italian baseball played by visually impaired and blind athletes is an adapted team sport which maintains the peculiar fast-moving features of this popular sport. It is also a mixed team game played together with sighted subjects. Here, we performed a national survey aimed at assessing the differences in psychological well-being (PWB) and quality of life (QoL) between visually impaired baseball players from Italian teams and non-players using a structured online questionnaire. Forty-three visually impaired baseball players and thirty-four visually impaired sedentary individuals completed a structured self-report survey including the validated 18-item Italian versions of the PWB (PWB-18) scale and the Short Form-12 (SF-12) questionnaire to assess the QoL. PWB-18 and SF-12 reference data from the Italian normally sighted population were also employed for comparison with the visually impaired baseball player group. Visually impaired baseball players reported better scores in all dimensions of the PWB-18 scale and significant higher scores in both physical and mental QoL evaluated by SF-12 than the non-player group. In addition, PWB-18 scale findings revealed significant differences between visually impaired baseball players and the reference normally sighted population consisting in lower scores for autonomy, environmental mastery, positive relations with others and purpose in life dimensions. Conversely, the mean scores for PWB-18 personal growth and self-acceptance dimensions were not significantly different between the two groups. The SF-12 questionnaire results demonstrated a significantly higher physical score in visually impaired players compared with the reference population. Instead, the SF-12 mental score of visually impaired athletes tended to be lower, though this difference was not statistically significant. Collectively, our findings suggest that the practice of Italian baseball may have a positive impact on PWB and QoL of visually impaired individuals.

## Introduction

Quality of life (QoL) is multidimensional and includes factors such as health, physical functioning, life satisfaction, sense of happiness and social well-being amongst others [1–3]. Increasing evidence supports the positive effects deriving from the participation in regular physical activity to achieve psychophysical health with an overall improvement of QoL [4]. In particular, regular physical activity is the mainstay of chronic disease prevention and health maintenance in both people with and those without disabilities, and clear evidence exists of its psychophysical benefits in visually impaired people [5–11]. Despite the importance of an active lifestyle, only a minority of the population is currently meeting the recommended levels of regular physical activity to achieve health and QoL benefits [12]. People with a disability, such as blind and visually impaired individuals, are on average even more inactive than the general population [10]. Visual impairment is one of the most common disabilities, and there are different levels of this dysfunction. ‘Low vision’, which refers to both moderate and severe visual impairments, and blindness collectively represent all visual impairments according to the International Classification of Diseases [13]. These conditions heavily compromise QoL and are associated with a decreased mobility. As a matter of fact, subjects with visual impairment have a more sedentary lifestyle respect to those with other disabilities [9]. In addition, the health-related fitness level of individuals with visual impairment results generally lower than that of sighted individuals [8, 9]. The absence of the sense of vision clearly can affect motor skills, though this does not prevent individuals who have a visual impairment from being physically active [3].

During the past decades, the adaptation of a variety of sports to subjects with disabilities and the ideation of new tailored sport activities have increased participation opportunities [3]. In particular, visually impaired people can participate in many recreational and sporting activities such as road cycling, swimming, sailing, snow skiing, goalball, equestrianism, athletics, archery, judo, torball, dance, showdown, fencing and 5-a-side football [3]. The practice of sports can ameliorate their sense of independence and autonomy, generating multiple physiological, sociological, and psychological benefits, and thus overcoming the disadvantages related to visual disability [3, 14, 15]. The positive impact of sport is also testified by a greater self-reliance [16] and the acquisition or improvement of motor skills [9, 17]. Visually impaired individuals who participate in regular sport activities may even improve their fitness reaching levels comparable to those of sighted subjects [9]. Of note, a recent research demonstrated that blind subjects playing torball have higher levels of socialization and better psychological well-being (PWB) compared to the non-player blind people [18]. However, studies taking into account both sport practice and QoL in blind and visually impaired individuals are scant [19].

In a previous preliminary investigation, we described for the first time the modality of Italian baseball game played by visually impaired subjects [17]. Briefly, Italian baseball for blind subjects is a mixed (males and females) team game played together with sighted subjects. This adapted baseball version, modified according to the visually impaired individual needs and ensuring the athlete’s safety, maintains the peculiar fast-moving characteristics of this popular sport. The positive effects of playing baseball on the balance of visually impaired athletes suggested that the peculiar features of this sport could enhance various motor skills potentially transferable into daily activities with an improvement of QoL [17]. On these premises, to determine whether playing baseball can improve the physical and mental well-being of people with visual impairment, here we performed a national survey based on a structured online questionnaire assessing the PWB and QoL of visually impaired baseball players from Italian teams in comparison with visually impaired sedentary individuals. In addition, we also compared PWB and QoL data from visually impaired baseball players with normative data from the Italian normally sighted population with the intent to further substantiate the role of baseball practice in mitigating the visual disability-related impact on psychophysical well-being. Finally, we investigated possible differences in PWB and QoL of visually impaired baseball players according to the level of visual disability.

## Materials and methods

### Study participants

Visually impaired baseball players from different Italian baseball teams competing at national level were invited to participate in an online survey questionnaire. In total, 43 (59.7%) of 72 visually impaired baseball players completed the questionnaire (Table 1). All baseball athletes had training for 2.5 hours/day once a week. Briefly, the Italian modified version of baseball for visually impaired people maintains the typical baseball chracteristics, though the field is modified to ensure the athlete safety (https://www.aibxc.it/giocoregole/index.php) [17]. Infact, the teams involved in the match alternate in the batting and defensive fields. Each team consists of 7 subjects: 5 visually impaired players and 2 sighted players. Of note, the visually impaired subjects must wear a blindfold so that there are no differences related to the different degrees of visual impairment [17]. The 2 sighted players have both a defensive role or serve as coaches at the second and third bases by beating the palettes to direct with sound the visually impaired players to the respective base. During the play, the batter player tosses the ball (regular size baseball with five holes and two sleigh bells inside) in the air and hits it. The ball must reach the defensive field of play (*i*.*e*. the left field beyond the line between the second and third base) after having bounced at least one time in the field. The blind batter runs around the sound-activated first base and attempts to reach the second and third bases assisted by the sighted coach. The blind runner is safe if reaching the bases before that a blind defensive player throws the ball to a sighted player positioned at the second base. The home plate is a long line that the runner must reach without any suggestion [17].

**Table 1:** List of Italian baseball teams composed of visually impaired athletes who responded to the survey.

Italian baseball teams	Region	Number of visually impaired baseball players (n = 43)
Patrini Malnate	Lombardy	2
Cvinta Ravenna	Emilia Romagna	2
Cagliari Tigers	Sardinia	3
Lampi Milano	Lombardy	6
Roma BXC	Lazio	5
Fiorentina BXC	Tuscany	5
Thunder’s Five Milano	Lombardy	7
Cagliari Thurpos	Sardinia	2
Umbria Redskins	Umbria	4
Staranzano Ducks	Friuli Venice Giulia	4
Leonessa Brescia	Lombardy	3

Thirty-four visually impaired sedentary individuals who completed an online survey questionnaire constituted the control group. This cross-sectional survey was conducted between January and February 2018. Study procedures were carried out following the rules of the Declaration of Helsinki of 1975 (https://www.wma.net/what-we-do/medical-ethics/declaration-of-helsinki/), revised in 2013. All subjects participated voluntarily and anonymously, and gave their informed consent. No ethics committee approval was needed for this anonymous online survey.

### Procedures

An online survey questionnaire was created using the Google Forms platform [20–22]. Invitation to participate in the study with a shareable link to access the survey and relevant instructions to answer the survey questions was sent by email to visually impaired athletes by the Italian Association of Baseball for Blind (AIBXC). Each participant had direct access to the online questionnaire using a Web browser and screen readers or screen magnification programs (*e*.*g*. Windows Jaws, Apple Voice Over) as computer softwares to assist the totally blind or visually impaired individuals in using a computer or a smartphone. Subject participation was voluntary and responses were completely anonymous and confidential. The online data were real time registered in the Google Survey database as Excel-type documents. The self-administered questionnaire contained closed questions with limited answer choice (see S1 File for the Italian and English versions of the administered questionnaire). Specifically, the survey consisted in a first part concerning sociodemographic characteristics including age, gender, educational status, job position and eventual clinical diagnosis of some diseases (*e*.*g*. myocardial infarction, angina pectoris, hypertension and diabetes). The second part of the survey collected information on the type and severity of visual disability (*i*.*e*. total blindness, severely sight-impaired and mildly sight-impaired according to the Classification of the International Blind Sports Federation) [18] and the practice of baseball and/or any other sports. Finally, the third part included the 18-item Italian versions of the PWB (PWB-18) scale and the Short Form-12 (SF-12) questionnaire to assess the PWB and QoL, respectively [23–30]. In particular, the PWB-18 scale, based on Ryff’s multidimensional model of PWB, evaluates the personal perception of well-being relative to 6 dimensions: autonomy, environmental mastery, personal growth, positive relations with others, purpose in life, and self-acceptance [31, 32]. This questionnaire is composed of 18 items (3 for each dimension) with a score ranging between 1 (strongly disagree) and 6 (strongly agree). A total PWB score was calculated by adding all six constructs. High single scores reflect a good self-acceptance, autonomy and environmental mastery as well as strong positive relations with others, purpose in life and personal growth, resulting in a high total score indicative of an overall positive PWB [23–26]. The PWB questionnaire reliability was assessed through the calculation of Cronbach's alpha coefficient. Since a coefficient value of 0.7 is considered indicative of an acceptable reliability [33], the calculated coefficient of 0.85 testified a good reliability of PWB questionnaire when administered to Italian visually impaired subjects. The Italian version of the SF-12 questionnaire consists of 2 components: a physical component score and a mental component score. Higher scores on these subscales indicate greater levels of functioning and a more favorable health status, thus being indicative of a better QoL [27]. Of note, the SF-12 questionnaire has previously been used to assess the health-related QoL in people with visual impairment [34, 35].

The questionnaire form for the control group differed in the second part of the survey that included only the questions on the type and severity of visual disability and participation or not in any physical or sport activities. Visually impaired individuals included in the sedentary control group were invited to participate in the survey by the Italian Institute for Research Training and Rehabilitation of visually impaired people (I.Ri.Fo.R. Onlus). A total of 65 subjects responded to the survey, but 31 were excluded because of participation in physical or sport activities. Therefore, the final study control group consisted of 34 visually impaired sedentary individuals.

### Statistical analysis

Data were entered using Microsoft Office Excel. All data are represented as mean ± SD or percentage. Statistical analysis was carried out using SPSS version 21.0. PWB-18 and SF-12 data from the baseball player group were compared either with sedentary control subjects or with normative data from the Italian normally sighted population [26, 27]. Differences between two groups were analyzed by unpaired Student’s *t-*test with post-hoc Bonferroni’s correction for multiple comparisons. One-way ANOVA followed by post-hoc Bonferroni’s test was used for comparisons of multiple subgroups of visually impaired baseball players. Adjusted p-values (p_adj_) <0.05 were considered statistically significant.

## Results

Out of 43 visually impaired baseball players (aged 38.1 ± 12 years) who took part in this study by answering the self-administered questionnaire, 36 (83.7%) were male and 7 (16.3%) female. The control group consisted of 34 visually impaired sedentary individuals (20 (58.8%) male and 14 (42.2%) female; mean age 42.2 ± 11.6 years) who did not practice any sport or physical activity in their leisure time. The characteristics of study participants are detailed in Table 2. No significant difference between the two groups was observed for the different variables taken into account (Table 2). Focusing on the group of visually impaired subjects playing baseball, 34 (79.1%) subjects were visually impaired from birth while 9 (20.9%) had acquired vision loss. In particular, 8 (88.9%) subjects were blind from over 10 years and 1 (11.1%) subject from 5 to 10 years. According to the classification of the International Blind Sports Federation (IBSA), B1 (blind) level was the most common type of reported visual disability (74.4%), followed by B2 (severely sight-impaired) (16.3%) and B3 (mildly sight-impaired) (9.3%) levels (Table 2). Information on the educational level, employment status and general health conditions collected by athlete interview is reported in Table 2. In particular, the educational level was classified into four categories based on the Italian school system, namely primary school, middle school, high school and university. Regarding the employment status, the majority of participants were employees (41.9%), followed by students (18.6%) and health professionals (11.6%). Moreover, 74.4% of athletes referred no pathology in addition to visual impairment. Forty (93%) baseball players had practiced other sports in the past. In addition, 23 (53.5%) athletes were practicing other sports at the time of the interview (*i*.*e*. torball, archey, swimming, athletics, showdown, skiing, fencing, 5-a-side football, judo, dancesport).

**Table 2:** Demographic data, description of visual disability and general health conditions of visually impaired subjects playing baseball and visually impaired sedentary individuals.

Variables	Baseball players (n = 43)	Sedentary individuals (n = 34)	p-value^#^
**Age** (years), mean ± SD (range)	38.1 ± 12 (15–60)	42.2 ± 11.6 (21–62)	NS
**Sex**, n (%)			
Male	36 (83.7)	20 (58.8)	0.03
Female	7 (16.3)	14 (42.2)	
**Blindness**, n (%)			
Congenital	34 (79.1)	22 (64.7)	NS
Acquired	9 (20.9)	12 (35.3)	
Classification of deficit^*^, n (%)			
B1	32 (74.4)	26 (76.5)	NS
B2	7 (16.3)	5 (14.7)	
B3	4 (9.3)	3 (8.8)	
**Educational level**, n (%)			
Primary school degree	1 (2.3)	2 (5.9)	NS
Middle school degree	13 (30.2)	8 (23.5)	
High school degree	22 (51.2)	12 (35.3)	
University	7 (16.3)	12 (35.3)	
Profession, occupation and job, n (%)			
Freelance	2 (4.7)	1 (2.9)	NS
Health profession	5 (11.6)	2 (5.9)	
Employee	18 (41.9)	12 (35.3)	
Worker	1 (2.3)	1 (2.9)	
Retiree	3 (7)	10 (29.5)	
Student	8 (18.6)	4 (11.8)	
Volunteering	2 (4.6)	1 (2.9)	
Unemployed	4 (9.3)	3 (8.8)	
**Health conditions**, n (%)			
Hypertension	4 (9.3)	5 (14.7)	NS
Diabetes	2 (4.6)	1 (2.9)	
Arthritis	3 (6.9)	4 (11.8)	
High cholesterol and triglycerides	3 (6.9)	5 (14.7)	
No disease	32 (74.4)	20 (58.8)	

B1 = from no light perception in both eyes to light perception, but inability to recognize the shape of a hand at any distance and in any direction (*i*.*e*. total blindness). B2 = from ability to recognize the shape of a hand to a visual acuity of 2/60 and/or visual field <5° (*i*.*e*. severely sight-impaired). B3 = from visual acuity above 2/60 to visual acuity of 6/60 and/or visual field >5° and <20° (*i*.*e*. mildly sight-impaired). NS, not significant

^*^Classification of the International Blind Sports Federation (IBSA).

^#^Student’s *t*-test for unpaired data.

**Fig 1:**
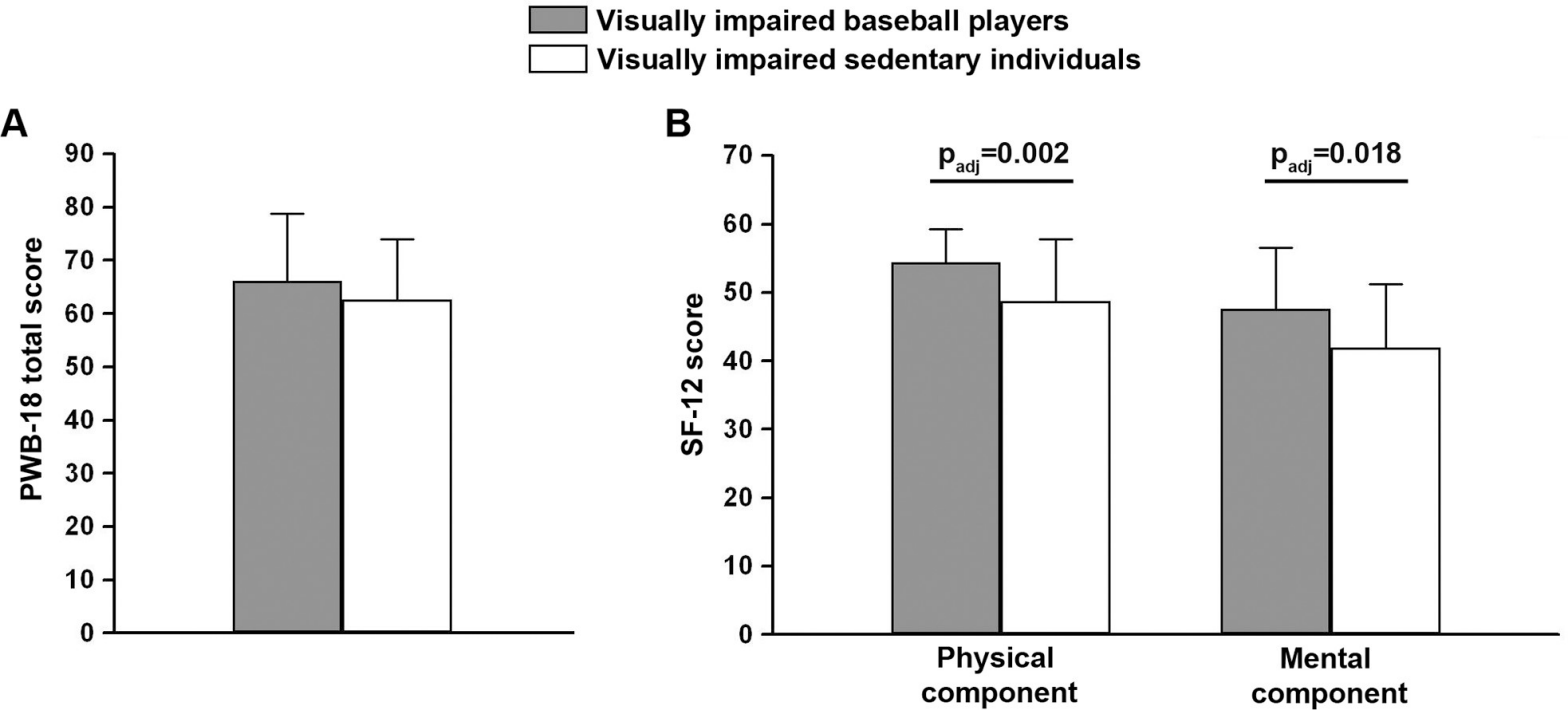
Comparison of PWB-18 total score (**A**) and SF-12 physical and mental component score (**B**) data from visually impaired baseball players and visually impaired sedentary individuals. Data are mean ± SD. Student’s *t*-test for unpaired data with post-hoc Bonferroni’s correction for multiple comparisons was used for statistical analysis; p_adj_, adjusted p-value.

Table 3 summarizes the results of PWB-18 and SF-12 questionnaires related to the visually impaired athletes playing baseball and sedentary controls. Of the athletes, 18 (41.9%) were playing baseball from at least 5 years, 7 (16.2%) from 3–5 years, and 12 (27.9%) from 1–3 years. In addition, 4 (9.3%) athletes were playing baseball from 6 months to 1 year, and only 2 athletes (4.7%) for at least 6 months. As expected, overall the sedentary group showed lower PWB-18 values than baseball players (Table 3). However, no significant differences in PWB-18 scale questionnaire score were found between visually impaired baseball players and visually impaired sedentary individuals (Table 3 and Fig 1A). In addition, SF-12 analysis revealed that baseball players had significantly higher scores for both physical and mental QoL compared with sedentary subjects (p_adj_ = 0.002 and p_adj_ = 0.018, respectively) (Table 3 and Fig 1B).

**Table 3:** Mean scores of psychological well-being scale and quality of life questionnaires in visually impaired baseball players compared with visually impaired sedentary individuals.

Variables	Baseball players	Sedentary individuals	p_adj_*
**Psychological Well-Being (PWB-18)**			
Autonomy	11.25 (3.12)	10.76 (2.42)	NS
Environmental mastery	10.74 (2.41)	10.67 (2.88)	NS
Personal growth	13.20 (2.54)	12.26 (3.48)	NS
Positive relations with others	9.34 (3.27)	9.07 (3.23)	NS
Purpose in life	10.04 (2.42)	8.82 (2.58)	NS
Self-acceptance	11.62 (2.81)	10.67 (2.79)	NS
Total score	66.23 (12.55)	62.67 (11.36)	NS
**Quality of Life (SF-12)**			
Physical component	54.38 (4.91)	48.77 (9.05)	0.002
Mental component	47.56 (8.92)	42.00 (9.25)	0.018

Values are mean (SD). NS, not significant.

*Student’s *t*-test for unpaired data with post-hoc Bonferroni’s correction for multiple comparisons; p_adj_, adjusted p-value.

**Fig 2:**
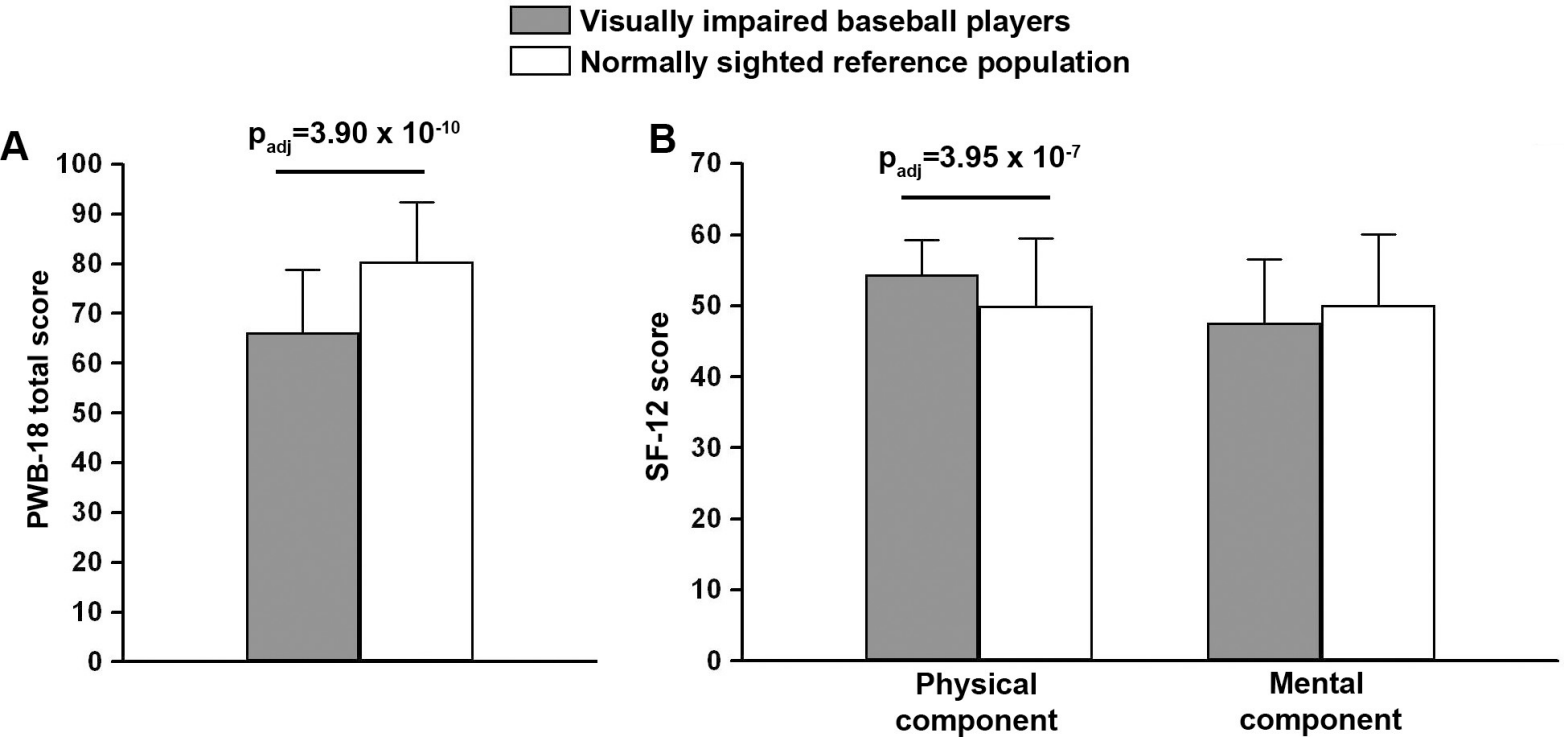
Comparison of PWB-18 total score (**A**) and SF-12 physical and mental component score (**B**) data from visually impaired baseball players and the Italian normally sighted reference population. Data are mean ± SD. Student’s *t*-test for unpaired data with post-hoc Bonferroni’s correction for multiple comparisons was used for statistical analysis; p_adj_, adjusted p-value.

Table 4 displays the comparison of score results of PWB-18 scale and SF-12 questionnaire between visually impaired baseball players and the Italian normally sighted control population. Concerning the PWB evaluation, visually impaired baseball players reported statistically significant lower values of almost all PWB-18 subscales compared with the Italian normally sighted controls (Table 4). Indeed, we detected lower mean scores also for PWB-18 personal growth and self-acceptance dimensions, though these differences did not reach the statistical significance (Table 4). As reported in Table 4 and displayed in Fig 2A, the PWB total score of the visually impaired baseball player group was significantly lower than that of Italian normally sighted controls (p_adj_ = 3.90 × 10^−10^). Data concerning the assessment of perceived health status using the SF-12 questionnaire are also displayed in Table 4. Visually impaired baseball players reported a significantly higher score in physical QoL compared with the normative data from the Italian normally sighted general population (p_adj_ = 3.95 × 10^−7^) (Table 4 and Fig 2B). Instead, the visually impaired athlete mental score tended to be lower, though this difference was not statistically significant (Table 4 and Fig 2B).

**Table 4:** Mean scores of psychological well-being scale and quality of life questionnaires in visually impaired baseball players compared with the Italian normally sighted population.

Variables	Baseball players	Italian reference population^§^	p_adj_*
**Psychological Well-Being (PWB-18)**			
Autonomy	11.25 (3.12)	14.33 (3.22)	8.91 × 10^−8^
Environmental mastery	10.74 (2.41)	14.31 (3.00)	1.51 × 10^−14^
Personal growth	13.20 (2.54)	14.16 (3.26)	NS
Positive relations with others	9.34 (3.27)	12.46 (3.23)	1.2 × 10^−7^
Purpose in life	10.04 (2.42)	12.5 (3.31)	2.54 × 10^−7^
Self-acceptance	11.62 (2.81)	12.53 (3.24)	NS
Total score	66.23 (12.55)	80.49 (11.99)	3.90 × 10^−10^
**Quality of Life (SF-12)**			
Physical component	54.38 (4.91)	50.03 (9.49)	3.95 × 10^−7^
Mental component	47.56 (8.92)	50.07 (9.98)	NS

Values are mean (SD). NS, not significant.

^**§**^PWB-18: Italian healthy workers [26]; SF-12: Italian general population [27].

*Student’s *t*-test for unpaired data with post-hoc Bonferroni’s correction for multiple comparisons; p_adj_, adjusted p-value.

Finally, the mean scores of PWB scale and QoL questionnaire for baseball players stratified according to visual disability level were lower in severely sight-impaired (B2 classification) than either blind or mildly sight-impaired (B1 and B3 classifications, respectively) subjects (Table 5). In particular, severely sight-impaired baseball players reported a statistically significant lower value of PWB-18 personal growth subscale compared with the blind athletes (p_adj_ = 0.026). Of note, the mildly sight-impaired athletes showed a significantly higher SF-12 mental score respect to blind athletes (p_adj_ = 0.035) (Table 5). Instead, no significant difference was found according to either gender or congenital/acquired visual disability. Similarly, no significant difference could be detected between baseball players practicing other sports and those who only practiced baseball.

**Table 5:** Mean scores of psychological well-being scale and quality of life questionnaires in visually impaired baseball players stratified according to the visual disability level.

Variables	B1 Baseball players	B2 Baseball players	B3 Baseball players*
**Psychological Well-Being (PWB-18)**			
Autonomy	11.21 (3.06)	11.14 (3.80)	11.75 (3.20)
Environmental mastery	11.03 (1.95)	9.42 (4.11)	10.75 (2.06)
Personal growth	13.84 (2.06)	10.85 (3.53)^#^	12.25 (1.70)
Positive relations with others	9.25 (3.18)	9.42 (4.23)	10 (2.94)
Purpose in life	10.25 (2.34)	9.14 (3.23)	10 (1.63)
Self-acceptance	11.62 (2.67)	10.71 (3.68)	13.25 (2.06)
Total score	67.21 (10.70)	60.71 (20.24)	68 (10.80)
**Quality of Life (SF-12)**			
Physical component	54.65 (4.26)	53.53 (7.33)	53.68 (6.31)
Mental component	46.77 (8.66)	45.33 (9.47)	57.74 (1.95)^§^

B1 = from no light perception in both eyes to light perception, but inability to recognize the shape of a hand at any distance and in any direction (*i*.*e*. total blindness). B2 = from ability to recognize the shape of a hand to a visual acuity of 2/60 and/or visual field <5° (*i*.*e*. severely sight-impaired). B3 = from visual acuity above 2/60 to visual acuity of 6/60 and/or visual field >5° and <20° (*i*.*e*. mildly sight-impaired). Values are mean (SD). Statistical analysis was performed by one-way ANOVA with post-hoc Bonferroni’s test.

^#^p_adj_ = 0.026 B2 Baseball players vs. B1 Baseball players.

^§^p_adj_ = 0.035 B3 Baseball players vs. B1 Baseball players.

*Classification of the International Blind Sports Federation (IBSA).

## Discussion

To the best of our knowledge, this is the first study assessing the PWB and QoL of visually impaired baseball players. Our findings demonstrate that baseball players with visual impairment have a better PWB than visually impaired non-sportive individuals. Similar results were reported in a previous study assessing the effect of playing Torball [18]. Moreover, we clearly detected a significantly greater QoL in the sportive group as testified by higher mean values of both SF-12 physical and mental scores in baseball players than in the sedentary group. Overall, these results highlight a positive relationship between the practice of baseball and PWB/QoL of visually impaired people.

To further strengthen our data, PWB-18 and SF-12 reference data from the Italian normally sighted population were also employed for comparison with the visually impaired baseball player group. As it could be expected due to the negative impact of visual impairment, PWB-18 scale findings revealed a significant difference between visually impaired baseball players and the Italian healthy working population consisting in lower scores for autonomy, environmental mastery, positive relations with others and purpose in life dimensions. Instead, the mean scores for PWB-18 personal growth and self-acceptance dimensions, though slightly lower, were not significantly different between the two groups. On the basis of these observations, we can speculate that the practice of baseball may positively influence the personal growth and self-acceptance of visually impaired subjects. Our data on QoL, which measures the perceived physical and mental health levels as well as the overall subjective well-being, seem also to support this hypothesis. Indeed, the SF-12 questionnaire results demonstrated a significantly higher physical score for visually impaired players compared with the Italian general population. Conversely, as it could be expected considering the psychological impact of visual disability, the SF-12 mental score of visually impaired athletes tended to be lower, though such a difference was not statistically significant.

In this context, it has been shown that visual disability affects QoL by limiting social interactions and independence [36]. In a recent study, a small group of blind people participating in torball has been shown to display higher levels of socialization and better PWB respect to blind non-players [18]. In addition, visually impaired student-athletes playing goalball may display a greater socialization respect to visually impaired non-athletes [37]. Here, we attempted to compare visually impaired subjects playing baseball with the general population in order to verify whether a team sport as baseball could improve PWB and QoL reaching levels similar to those of normally sighted people. Of note, the general population consists of either physically active or inactive subjects, and this could explain the higher level of physical well-being reported by visually impaired people playing baseball. PWB and QoL are dynamic constructs and can be affected by various social, cultural and environmental factors at any particular moment of an individual’s experience [38]. It is also widely acknowledged that the health benefits of participation in physical activity are not limited to physical well-being but also incorporate mental health. In fact, physical activity clearly contributes to PWB enhancing mood and relieving stress, as well as improving self-confidence and self-acceptance [15, 18, 39]. Physical activity and sports also represent a viable strategy for improving the QoL defined as a personal sense of physical and mental health, social functioning and emotional well-being [15, 18, 39]. More specifically, there is evidence that participation in team sports rather than individual activities may be associated with better health outcomes, due to the social nature of the participation [40]. In particular, many different psychological and social health benefits have been reported, the most common being an improvement in self-esteem and social interaction followed by fewer depressive symptoms [40]. Leisure time physical activity and participation in sports may help to overcome disability-related psychological fears, resulting in better self-esteem, self-confidence and self-competence that are essential to increase social skills [39]. Given its peculiar features, the practice of Italian baseball may improve the processes of socialization and integration ameliorating the overall QoL of visually impaired individuals. Of note, baseball is an outdoor sport mainly practiced during the good season, and during winter season the visually impaired people often practice other sports to maintain their fitness. However, when comparing the PWB and QoL data of baseball players practicing other sports with those who only practiced baseball we could not find any significant difference.

As far as differences related to the levels of visual disability are concerned, in line with literature data [19], mildly sight-impaired individuals reported better QoL than either blind or severely sight-impaired individuals mainly in the SF-12 mental score. Moreover, it is noteworthy that all mean scores of PWB scale and QoL questionnaire were lower in severely sight-impaired subjects than in either blind or mildly sight-impaired subjects. These data may be consistent with the notion that the main difference between the blind and the severely sight-impaired subjects is that the former see their blindness as their characteristic trait, while the latter try to function as if they have normal sight [19]. Therefore, it can be hypothesized that the slight visual residue of severely sight-impaired subjects might represent a self barrier to the improvement of social skills and socialization level with consequent negative impact on PWB and QoL.

Overall, these findings should be interpreted in the context of the limitations of the present study. Indeed, our data may be limited by the relatively small sample of visually impaired participants, especially for the analyses of baseball players stratified according to the visual disability level, as well as the study design based on self-report assessments. However, health psychology research studies are mainly based on data collected through questionnaires or surveys [41]. In addition, considering the cross-sectional nature of our study, further longitudinal investigations are warranted. Finally, we cannot exclude the possibility that baseball practice might have attracted mainly visually impaired subjects with greater psychophysical ability, thus resulting in a partial athlete self-selection within the study group.

## Conclusions

In summary, despite the aforementioned limitations, the herein reported positive PWB and QoL outcomes suggest that visually impaired individuals could reach a personal growth and improve their self-acceptance, purpose in life and social skills through the practice of Italian baseball. Given its peculiar features, baseball participation might indeed favor an increase in self-esteem, social contacts and PWB, with consequent improvement of QoL of visually impaired people. Therefore, we believe that subjects with visual impairment should be encouraged to play this team sport. Our study may also contribute to promote and popularize worldwide the practice of Italian baseball for visually impaired subjects.

## Supporting information

S1 File Italian and English versions of the administered questionnaire. (PDF)
