# Supplementary material for: Psychological well-being and quality of life in visually impaired baseball players: An Italian national survey
Source: PLoS One. 2019 Jun 6;14(6):e0218124. doi: 10.1371/journal.pone.0218124 (PMC6553783; doi:10.1371/journal.pone.0218124)
Supplement: S1 File — (PDF) [file pone.0218124.s001.pdf]

S1 File. Italian and English versions of the administered questionnaire.

ITALIAN version

Questionario

Nome___________________

Lettera iniziale del Cognome____________________

Sesso ☐ maschio ☐ femmina

Data di nascita________________

Titolo di studio?

- ☐ Nessuno
- ☐ Elementare
- ☐ Licenza media Superiore
- ☐ Diploma/Maturità
- ☐ Laurea

Professione?

- ☐ Libero professionista
- ☐ Professione Sanitaria
- ☐ Impiegato
- ☐ Operaio
- ☐ Casalinga
- ☐ Pensionato
- ☐ Studente
- ☐ Disoccupato
- ☐ Altro

La sua disabilità visiva è?

- ☐ Congenita
- ☐ Acquisita

Se la disabilità è acquisita, indicare il tempo:

- ☐ Da meno di un anno
- ☐ Da un anno a tre anni
- ☐ Da tre anni a cinque anni
- ☐ Da cinque a dieci anni
- ☐ Oltre 10 anni

Da quanto tempo pratica il baseball?

- ☐ Da almeno sei mesi
- ☐ Da sei mesi ad un anno
- ☐ Da un anno a tre anni
- ☐ Da tre a cinque anni
- ☐ Oltre i cinque anni

In quale squadra gioca?

____

Classificazione del suo deficit visivo?

- ☐ Cieco assoluto
- ☐ Ipovedente grave
- ☐ Ipovedente lieve

Pratica attualmente altri sports?

- ☐ Si
- ☐ No

Se Si, quali sport pratica?

- ☐ Torball
- ☐ Tiro con l'arco
- ☐ Nuoto
- ☐ Atletica
- ☐ Judo
- ☐ Calcio a 5
- ☐ Showdown
- ☐ Danza sportiva
- ☐ Sci
- ☐ Scherma
- ☐ Altro

Ha praticato sport in passato?

- ☐ Si
- ☐ No

Se Si, quale sport?

- ☐ Torball
- ☐ Tiro con l'arco
- ☐ Nuoto
- ☐ Atletica
- ☐ Judo
- ☐ Calcio a 5
- ☐ Showdown
- ☐ Danza sportiva
- ☐ Sci
- ☐ Scherma
- ☐ Altro

Segni se un medico le ha mai diagnosticato le seguenti malattie:

- ☐ Infarto/Angina pectoris
- ☐ Pressione alta
- ☐ Malattie respiratorie
- ☐ Artrosi
- ☐ Diabete
- ☐ Colesterolo o trigligeridi alti
- ☐ Nessuna

Scala PWB-18

Legga attentamente ciascuna domanda e segni quanto ciascuna affermazione è vera per Lei utilizzando una scala da 1 (non è il mio caso) a 6 (è proprio il mio caso).

	Non è il mio caso 1	2	3	4	5	È proprio il mio caso 6
1. Tendo ad essere influenzato dalle persone che hanno una forte personalità						
2. Ho fiducia nelle mie opinioni anche se sono contrarie a quelle degli altri						
3. Giudico me stesso secondo quello che penso sia importante e non per ciò che gli altri pensano sia importante						
4. In generale ho la sensazione di padroneggiare la situazione in cui mi trovo						
5. Le richieste della vita di tutti i giorni spesso mi abbattono						
6. Sono molto bravo a gestire le molte responsabilità della vita quotidiana						
7. Penso sia importante avere nuove esperienze che ti aiutano a confrontare l'opinione che hai di te e del mondo circostante						
8. Per me la vita è stato un continuo processo di apprendimento, cambiamento e crescita						
9. Ho rinunciato a grossi miglioramenti o cambiamenti nella mia vita già da molto tempo						
10. Mantenere relazioni stabili è stato per me difficile e frustrante						
11. La gente mi descriverebbe come una persona disponibile, pronta a condividere il mio tempo con gli altri						
12. Non ho avuto esperienza di molte relazioni calorose e di fiducia con gli altri						
13. Vivo la vita giorno per giorno e non penso al futuro						
14. Alcune persone sono senza progetti, ma io non sono una di quelle						
15. A volte ho la sensazione di aver già fatto tutto quello che si poteva fare						
16. Sono soddisfatto di come sono andate le cose nella mia vita						
17. Sono soddisfatto della maggior parte degli aspetti della mia personalità						
18. Molte volte non mi sento soddisfatto dei risultati ottenuti nella mia vita						

SF-12

Legga attentamente ciascuna domanda e segni l'affermazione più vera per Lei.

1. In generale, direbbe che la Sua salute è
Eccellente……................. 1
Molto buona................................ 2
Buona………................................... 3
Passabile.. 4
Scadente... 5

2. La sua salute La limita attualmente nello svolgimento di attività di moderato impegno fisico (come spostare un tavolo, usare l'aspirapolvere, giocare a bocce o fare un giro in bicicletta, ecc.)?
SI, mi limita parecchio…………. 1
SI, mi limita parzialmente……………. 2
NO, non mi limita per nulla..………………. 3

3. La sua salute La limita attualmente nel salire qualche piano di scale ?
SI, mi limita parecchio…………. 1
SI, mi limita parzialmente……………. 2
NO, non mi limita per nulla..………………. 3

4. Nelle ultime 4 settimane, ha reso meno di quanto avrebbe voluto sul lavoro o nelle altre attività quotidiane, a causa della sua salute fisica?
SI…………. 1
NO….……………. 2

5. Nelle ultime 4 settimane, ha dovuto limitare alcuni tipi di
lavoro o di altre attività, a causa della sua salute fisica?
SI…………. 1
NO ….……………. 2

6. Nelle ultime 4 settimane, ha reso meno di quanto avrebbe voluto sul lavoro o nelle altre attività quotidiane, a causa del suo stato emotivo (quale il sentirsi depresso o ansioso)?
SI…………. 1
NO ….……………. 2

7. Nelle ultime 4 settimane, ha avuto un calo di concentrazione sul lavoro o nelle altre attività quotidiane, a causa del suo stato emotivo (quale il sentirsi depresso o ansioso)?
SI…………. 1
NO….……………. 2

8. Nelle ultime 4 settimane, in che misura il dolore l'ha ostacolata nel lavoro che svolge abitualmente (sia in casa sia fuori casa)?
Per nulla…………. 1
Molto poco.……………. 2
Un po'……...………………. 3
Molto………………....……………. 4
Moltissimo.….. ………………………. 5

9. Per quanto tempo nelle ultime 4 settimane si è sentito calmo e sereno?
Sempre…………. 1
Quasi sempre………. 2
Molto tempo……………….3
Una parte del tempo…………. 4
Quasi mai... ……………………..… 5
Mai………………………………….…… 6

10. Per quanto tempo nelle ultime 4 settimane si è sentito pieno di energia?
Sempre…………. 1
Quasi sempre………. 2
Molto tempo……………….3
Una parte del tempo…………. 4
Quasi mai.…………………….…… 5
Mai………………………………….…… 6

11. Per quanto tempo nelle ultime 4 settimane si è sentito scoraggiato e triste?
Sempre…………. 1
Quasi sempre………. 2
Molto tempo……………… 3
Una parte del tempo…………. 4
Quasi mai……………………….… 5
Mai……………..……………………..… 6

12. Nelle ultime 4 settimane, per quanto tempo la Sua salute fisica o il Suo stato emotivo hanno interferito nelle sue attività sociali, in famiglia, con gli amici?
Sempre…………. 1
Quasi sempre………. 2
Molto tempo……………… 3
Una parte del tempo…………. 4
Quasi mai……………………….… 5
Mai……………..……………………..… 6

ENGLISH version

Questionnaire

Firts name___________________

Surname initial letter________________

Gender ☐ male ☐ female

Date of birth ________________

Educational level?

- ☐ No study
- ☐ Primary school
- ☐ Middle school
- ☐ High school
- ☐ University

Occupation?

- ☐ Freelance
- ☐ Health professional
- ☐ Employee
- ☐ Worker
- ☐ Housewife
- ☐ Retired
- ☐ Student
- ☐ Unemployed
- ☐ Other

Your visual impairment is?

- ☐ Congenital
- ☐ Acquired

If the visual impairment is acquired, indicate the time

- ☐ Less than one year
- ☐ From one year to three years
- ☐ From three years to five years
- ☐ From five years to ten years
- ☐ Over ten years

How long have you been playing baseball?

- ☐ at least six months
- ☐ six months to one year
- ☐ one year to three years
- ☐ three to five years
- ☐ over five years

Baseball team?

____

Your visual impairment classification?

- ☐ Blind
- ☐ Severely sight-impaired
- ☐ Mildly sight-impaired

Do you currently practice other sports?

- ☐ Yes
- ☐ No

If you answered yes, which sport?

- ☐ Torball
- ☐ Archery
- ☐ Swimming
- ☐ Athletics
- ☐ Judo
- ☐ 5-a-side football
- ☐ Showdown
- ☐ Dance sport
- ☐ Ski
- ☐ Fencing
- ☐ Other

In the past, did you practice sports?

- ☐ Yes
- ☐ No

If you answered yes, which sport?

- ☐ Torball
- ☐ Archery
- ☐ Swimming
- ☐ Athletics
- ☐ Judo
- ☐ 5-a-side football
- ☐ Showdown
- ☐ Dancesport
- ☐ Skiing
- ☐ Fencing
- ☐ Other

Has a doctor ever diagnosed the following diseases?

- ☐ Infarct/Angina pectoris
- ☐ Hypertension
- ☐ Respiratory diseases
- ☐ Arthritis
- ☐ Diabetes
- ☐ High cholesterol and triglycerides
- ☐ No disease

PWB-18 SCALE

Read each question and sign carefully to which extent each statement is proper for you. Using a scale of 1 (strongly disagree) to 6 (strongly agree).

	Strongly disagree 1	2	3	4	5	Strongly agree 6
1. I tend to be influenced by people who have strong personality						
2. I have confidence in my own opinions even if they are opposed to those of others						
3. I judge myself by what I think is important and not by what others think is important						
4. In general, I feel I am in charge of the situation in which I live						
5. The requests of everyday life often bring me down						
6. I am very good at managing the many responsibilities of everyday life						
7. I think it is important to have new experiences that help you to compare your opinion of yourself and the world around you						
8. For me life has been a continuous process of learning, change and growth						
9. I gave up trying to make big improvements or changes in my life a long time ago						
10. For me has been difficult and frustrating to maintain stable relationships						
11. People would describe me as an available person, ready to share my time with others						
12. I have not experienced many warm and trusting relationships with others						
13. I live life one day at a time and don't really think about the future						
14. Some people have no plans, but I am not one of them						
15. Sometimes I have the feeling that I have already done everything that could be done						
16. I am pleased with how things have turned out in my life						
17. I like most aspects of my personality						
18. Many times I am not satisfied with the results achieved in my life						

SF-12

Read each question and sign carefully which statement is proper for you.

1. In general, would you say your health is?
Excellent.......................... 1
Very Good.................................. 2
Good... 3
Fair... 4
Poor.. 5

2. Does your health now limit you during moderate physical activities (such as moving a table, pusshing a vacuum cleaner, bowling or cycling, etc.)?
Yes, limited a lot............ 1
Yes, limited a little................ 2
No, not limited at all.................... 3

3. Does your health now limit you during climbing a few flights of stairs?
Yes, limited a lot............ 1
Yes, limited a little................ 2
No, not limited at all.................... 3

4. During the past 4 weeks, have you accomplished less than you would like with your work or other regular daily activities as a result of your physical health?
Yes............ 1
No................... 2

5. During the past 4 weeks, did you have to limit certain types of work or other activities as a result of your physical health?
Yes............ 1
No 2

6. During the past 4 weeks, have you accomplished less than you would like with your work or other regular daily activities as a result of any emotional problem (such as feeling depressed or anxious) ?
Yes............ 1
No 2

7. During the past 4 weeks, did you have a concentration decline at work or in other activities as a result of any emotional problem (such as feeling depressed or anxious) ?
Yes............ 1
No................... 2

8. During the past 4 weeks, how much did pain interfere with your normal work (including both work outside home and housework)?
Not at all............ 1
A little bit................ 2
Moderately.............................. 3
Quite a bit 4
Extremely.................................. 5

9. How much of the time during the past 4 weeks, have you felt calm and peaceful?

All of the time 1
Most of the time.......... 2
A good bit of the time 3
Some of the time............ 4
A little of the time............................... 5
None of the time 6

10. How much of the time during the past 4 weeks, have you felt full of energy?

All of the time 1
Most of the time.......... 2
A good bit of the time 3
Some of the time............ 4
A little of the time............................... 5
None of the time 6

11. How much of the time during the past 4 weeks, have you felt downhearted and sad?

All of the time 1
Most of the time.......... 2
A good bit of the time 3
Some of the time............ 4
A little of the time... 5
None of the time 6

12. During the past 4 weeks, how much of the time has your physical health or emotional status interfered with your social activities (for example with friends, relatives, etc.)?

All of the time 1
Most of the time.......... 2
A good bit of the time 3
Some of the time............ 4
A little of the time............................... 5
None of the time 6
